# Supplementary material for: Role and mechanism of NCAPD3 in promoting malignant behaviors in gastric cancer
Source: Front Pharmacol. 2024 Apr 22;15:1341039. doi: 10.3389/fphar.2024.1341039 (PMC11070777; doi:10.3389/fphar.2024.1341039)
Supplement: Supplementary file 11 [file DataSheet2.ZIP › GSEA/Oncogenic signature/my_analysis.Gsea.1599461962066/ATF2_UP.V1_DN.html]

Details for gene set ATF2\_UP.V1\_DN[GSEA]

|  || Dataset | filtered\_dataset.sample\_info.cls#WT\_versus\_NCAPD3\_MUT |
| Phenotype | sample\_info.cls#WT\_versus\_NCAPD3\_MUT |
| Upregulated in class | NCAPD3\_MUT |
| GeneSet | ATF2\_UP.V1\_DN |
| Enrichment Score (ES) | -0.44852656 |
| Normalized Enrichment Score (NES) | -1.9335232 |
| Nominal p-value | 0.0 |
| FDR q-value | 0.031469062 |
| FWER p-Value | 0.203 |
Table: GSEA Results Summary

  

Fig 1: Enrichment plot: ATF2\_UP.V1\_DN      
 Profile of the Running ES Score & Positions of GeneSet Members on the Rank Ordered List

  

| SYMBOL | TITLE | RANK IN GENE LIST | RANK METRIC SCORE | RUNNING ES | CORE ENRICHMENT || 1 | 51279 | C1RL | 31 | 0.991 | 0.0703 | No |
| 2 | 22834 | ZNF652 | 164 | 0.711 | 0.0419 | No |
| 3 | 4091 | SMAD6 | 519 | 0.462 | -0.1689 | No |
| 4 | 9653 | HS2ST1 | 591 | 0.429 | -0.1798 | No |
| 5 | 31 | ACACA | 780 | 0.334 | -0.2836 | No |
| 6 | 5292 | PIM1 | 885 | -0.300 | -0.3301 | No |
| 7 | 51704 | GPRC5B | 1043 | -0.438 | -0.4018 | Yes |
| 8 | 3556 | IL1RAP | 1099 | -0.476 | -0.3969 | Yes |
| 9 | 353511 | PKD1P6 | 1172 | -0.539 | -0.3982 | Yes |
| 10 | 6038 | RNASE4 | 1194 | -0.570 | -0.3601 | Yes |
| 11 | 8863 | PER3 | 1227 | -0.601 | -0.3269 | Yes |
| 12 | 80201 | HKDC1 | 1289 | -0.681 | -0.3072 | Yes |
| 13 | 885 | CCK | 1302 | -0.703 | -0.2502 | Yes |
| 14 | 90865 | IL33 | 1325 | -0.745 | -0.1964 | Yes |
| 15 | 1116 | CHI3L1 | 1339 | -0.777 | -0.1332 | Yes |
| 16 | 4919 | ROR1 | 1385 | -0.954 | -0.0765 | Yes |
| 17 | 1491 | CTH | 1391 | -1.004 | 0.0136 | Yes |
Table: GSEA details [plain text format]

  

Fig 2: ATF2\_UP.V1\_DN      
 Blue-Pink O' Gram in the Space of the Analyzed GeneSet

  

Fig 3: ATF2\_UP.V1\_DN: Random ES distribution      
 Gene set null distribution of ES for **ATF2\_UP.V1\_DN**
